# Whole genome sequencing and taxonomic profiling of two *Pantoea* sp. isolated from environmental samples in Israel

**DOI:** 10.1186/s12863-022-01049-7

**Published:** 2022-04-21

**Authors:** Yehoudit Guedj-Dana, Inbar Cohen-Gihon, Ofir Israeli, Ohad Shifman, Tamar Aminov, Shahar Rotem, Raphael Ber, Anat Zvi

**Affiliations:** Department of Biochemistry and Molecular Genetics, Israel Institute for Biological Research, Ness Ziona, Israel

**Keywords:** *Pantoea*, Environmental samples, Whole genome sequencing, Oxford nanopore, De novo assembly, Taxonomy

## Abstract

**Objective:**

As part of a research aiming at the isolation of bacteria secreting growth inhibiting compounds, cultures of *Francisella tularensis* were implanted in environmental samples and monitored for inhibition zones on agar. Two antibiotic-like secreting bacteria were isolated, their genomic sequence was deciphered and taxonomic profiling analysis classified them as belonging to the *Pantoea* genus.

**Data description:**

Two bacterial isolates exhibiting growth inhibition zones to *F. tularensis* (LVS) were analyzed using the Oxford Nanopore Technology (ONT). Preliminary de novo assembly of the reads was performed, followed by taxonomic profiling based on Multi Locus Sequence Analysis (MLSA) and implementation of the Average Nucleotide Identity (ANI) measure. The genomic sequences resulted in the identification of two different *Pantoea* species, denoted EnvD and EnvH.

Subsequent de novo genome assembly generated 5 and 10 contigs for EnvD and EnvH, respectively. The largest contig (4,008,183 bps and 3,740,753 bps for EnvD and EnvH, respectively), overlaps to a major extent to the chromosome of closely related *Pantoea* species. ANI values calculated for both isolates revealed two apparently new species of the *Pantoea* genus.

Our study deciphered the identity of two bacteria producing antibiotic-like compounds, and the genomic sequence revealed they represent distinct *Pantoea* species.

## Objective

*Pantoea* is a genus of Gram-negative bacteria belonging to the family Erwiniaceae. These bacteria were isolated from a multitude of environmental sources (such as plants, animals, water, soil and human). They are distributed in nature, and over 25 species were already documented [1–4]. In this study we present the taxonomic profiling of two environmental isolates which were identified by their ability to inhibit growth of *F. tularensis* (LVS) colonies. The isolates were sequenced by long read sequencing and assembled by a de novo assembly. We calculated the ANI measure to establish the species relatedness and the MLSA method was implemented to generate a phylogenetic tree showing these variants as new *Pantoea* species.

## Data description

In this study, over 20 environmental samples were collected from distinct different geographic locations in Israel (natural sources). The samples were prepared by wiping a 20 cm^2^ area of urban asphalt roads with 3 sterile cotton swabs (Copan Italia SPA) moistened aseptically in sterile phosphate-buffered saline (PBS) solution. The swabs were discarded after extraction by vortex in 5 mL PBS. After 2 min. of passive sedimentation of dust and dirt, 4.2 mL were transferred to new sterile tube. An aliquot of 0.1 mL of each environmental sample was mixed with 0.1 mL of PBS containing 10^8^ cfu/ml of indicator bacteria (*F. tularensis* LVS), the mix was spread on cysteine heart agar with hemoglobin (CHA, Difco), plates were incubated for 2 days at 37 °C and monitored for colonies demonstrating inhibition zone for the indicator strain. Several such colonies were further isolated to pure cultures on CHA, and their inhibition to LVS was verified. Two of the isolates showed differential capability to grow in Brain Heart Infusion broth, and were denoted herein as EnvD (from Talpiot industrial zone, Jerusalem) and EnvH (from Romema neighborhood, Haifa). For DNA purification, EnvD and EnvH colonies were grown on CHA, suspended to high density in 1 mL PBS, mixed 1:1 with ATL buffer, heated at 100 °C for 30 min before DNA purification using the QIAamp DNA blood minikit (Qiagen). Whole genome sequencing was conducted in GenoHub facility (https://genohub.com) using the Oxford Nanopore MinION Technology. Libraries for both EnvD and EnvH were prepared using the SQK-LSK109 ligation sequencing kit (Oxford Nanopore Technologies) and sequencing was conducted using an R9 flow cell. Basecalling was performed using Guppy v 4.4.2. A total of 210,000 reads for EnvD (mean average length of 3854 bps) and 190,000 reads for EnvH (mean average length of 1881 bps) were obtained, resulting in a coverage of 155x and 89x, respectively (data file 1) [5].

De novo assembly was conducted by implementing Flye [6], designed for the assembly of long reads generated by ONT. Five contigs were obtained for EnvD, the largest harboring a length of 4,008,183 bps, the N50 value being therefore 4,008,183. The EnvH generated reads were assembled to a total of 10 contigs, the longest being 3,740,753 bps (which is therefore the N50 value). A rough and preliminary estimation of the taxonomical relatedness of the two isolates conducted by a Blast analysis [7] against the nucleotide database (nt, https://www.ncbi.nlm.nih.gov/nucleotide/), disclosed similarity to the *Pantoea* genus. Subsequently, the longest contig of each isolate was compared to all publicly available *Pantoea* sequences (National Center for Biotechnology Information, NCBI), revealing that the best matching hits for EnvD and EnvH are *Pantoea agglomerans* and *Pantoea stewartii*, respectively. Accordingly, the contigs generated by the assembly were aligned to their closest species, using Mauve [8]. The alignments display a nearly complete coverage of the reference chromosome by the largest contig, both for EnvD and EnvH. In addition, two of the remaining contigs overlap plasmid regions in the reference genomes (data file 2) [9].

To further characterize whether the sequenced genomes can be associated with one of the already known *Pantoea* species, we used the ANI measure as a well-established whole genome similarity metrics [10–14]. The ANI value was estimated using the FastANI algorithm [12]. Pairwise ANI values of at most 82% were obtained for each of the sequenced genomes with genomes representing known *Pantoea* species [15]. According to the generally accepted cutoff value of 95% used as a boundary for species delineation [14, 16], it appears that EnvD and EnvH constitute new species within the *Pantoea* genus. To note, the pairwise ANI value between EnvD and EnvH is 83%, therefore representing two distinct lineages.

To assign the taxonomic profiling of the two isolates, we implemented the Multi Locus Sequence Analysis (MLSA) typing method, tailored for phylogeny analysis of *Pantoea* species [17, 18], using five core genes that are effective at species-level delineation of the genus *Pantoea*: fusA*, *gyrB*, *leuS*, *pyrG and rpoB [1]. The sequence of the genes orthologous to EnvD and EnvH were extracted from the contig sequences covering the chromosome regions (contig 4 for EnvD and contig 3 for EnvH), and concatenated into a mini-gene. Alongside, the five protein-coding genes were extracted from 37 representative, reference and/or type strains of *Pantoea* and *Tatumella* species (for a complete list of species included in the set of 37 genomes, refer to [17]). The multiple alignment of the 37 concatenated sequences together with the concatenated sequences originating from EnvD and EnvH was constructed using the MAFFT algorithm (Multiple Alignment using fast Fourier Transform) [19] of the MegAlign™ Pro (©1993–2020) (DNASTAR®). Phylogenetic analysis of the aligned sequences was conducted using the PhyML tool [20], which estimates Maximum-Likelihood phylogenies (NGPhylogeny.fr [21]). Each of the new isolates, EnvD and EnvH, forms a distinct and separate branch (data file 3) [22]. While EnvH is related to *Pantoea stewartii*, *Pantoea ananatis* and *Pantoea allii* species, EnvD is not only branching from a separate cluster, but is also relatively distant from species in this cluster (which includes, among others, *Pantoea agglomerans*, corroborating with the preliminary assignment described above for EnvD).

To conclude, two growth-inhibiting bacteria from environmental samples collected from two distinct areas in Israel were identified and assigned as belonging to the *Pantoea* genus. Their taxonomical profiling unveiled that these bacteria can be classified as new *Pantoea* species diverging from known *Pantoea* species described up to date. Further experimental characterization of the mechanism involved in the LVS growth inhibition of these *Pantoea* is now undertaken. Please see Table 1 for links to Data files 1-4 and Data sets 1-3. 1.

**Table 1:** Overview of data files/data sets

Label	Name of data file/data sets	File types (file extension)	Data repository and identifier (DOI or accession number)
Data file 1	Sequencing and assembly metrics	Portable Document Format file (.pdf)	https://doi.org/10.6084/m9.figshare.15111765.v1 [5]
Data file 2	Alignment of contigs to a reference genome	Portable Document Format file (.pdf)	https://doi.org/10.6084/m9.figshare.15105198.v1 [9]
Data file 3	ANI values for *Pantoea* sp. EnvD and *Pantoea* sp. EnvH	Portable Document Format file (.pdf)	https://doi.org/10.6084/m9.figshare.19204658.v2 [15]
Data file 4	Phylogenetics analyses	Portable Document Format file (.pdf)	https://doi.org/10.6084/m9.figshare.15111588.v3 [22]
Data set 1	Sequencing reads of *Pantoea* sp. EnvD and *Pantoea* sp. EnvH	Fastq file (.fastq.gz)	SRP316834 [23]
Data set 2	Genome assembly of *Pantoea* sp. EnvD	FASTA / GenBank / ASN.1	JAGTWO000000000.1 [24]
Data set 3	Genome assembly of *Pantoea* sp. EnvH	FASTA / GenBank / ASN.1	JAGTWN000000000.1 [25]

## Limitations

- The de novo assembly resulted in a number of contigs; while the longest contig very nearly cover the chromosome region, some other regions of the EnvD and EnvH sequenced genomes are fragmentally covered.
- By reason of practical considerations, the phylogeny relatedness of the two *Pantoea* sp. EnvD and EnvH is based on a limited dataset of 37 known *Pantoea* species, comprising of representative, reference and/or type strains of *Pantoea* and *Tatumella* species.

## Data Availability

Data files 1–4 described in this Data note can be freely and openly accessible on Figshare (https://figshare.com/) [5, 9, 22]. Data sets 1–3 are available on the NCBI database. The raw reads have been submitted to the NCBI Sequence Read Archive under the accession number SRP316834 for *Pantoea* sp. EnvD and *Pantoea* sp. EnvH (Data set 1) [23–25]. The genome assemblies of the two samples were submitted to NCBI GenBank and are available under the accession number JAGTWO000000000 for the *Pantoea* sp. EnvD and the accession number JAGTWN000000000 for the *Pantoea* sp. EnvH (Data sets 2–3) [24, 25].

## Abbreviations

MLSA: Multi Locus Sequence Analysis
ANI: Average Nucleotide Identity
ONT: Oxford Nanopore Technologies
MAFFT: Multiple Alignment using fast Fourier Transform
PhyML: Phylogeny Maximum-Likelihood
